# The impact of IL-1 modulation on the development of lipopolysaccharide-induced cognitive dysfunction

**DOI:** 10.1186/cc9019

**Published:** 2010-05-14

**Authors:** Niccolò Terrando, António Rei Fidalgo, Marcela Vizcaychipi, Mario Cibelli, Daqing Ma, Claudia Monaco, Marc Feldmann, Mervyn Maze

**Affiliations:** 1 Department of Anesthetics, Pain Medicine and Intensive Care, Imperial College London, Chelsea & Westminster Hospital, 369 Fulham Road, London, SW10 9NH, UK; 2 Department of Anesthesia and Perioperative Care, UCSF, 521 Parnassus Avenue, San Francisco, CA 94143-0648, USA; 3 Kennedy Institute of Rheumatology, Faculty of Medicine, Imperial College London, 65 Aspenlea Road, London W6 8LH, UK; 4 Department of Anesthesia, St. George's Hospital, Blackshaw Road, London SW17 0QT, UK

## Abstract

**Introduction:**

The impact of pro-inflammatory cytokines on neuroinflammation and cognitive function after lipopolysaccharide (LPS) challenge remains elusive. Herein we provide evidence that there is a temporal correlation between high-mobility group box 1 (HMGB-1), microglial activation, and cognitive dysfunction. Disabling the interleukin (IL)-1 signaling pathway is sufficient to reduce inflammation and ameliorate the disability.

**Methods:**

Endotoxemia was induced in wild-type and IL-1R^-/- ^mice by intra peritoneal injection of *E. Coli *LPS (1 mg/kg). Markers of inflammation were assessed both peripherally and centrally, and correlated to behavioral outcome using trace fear conditioning.

**Results:**

Increase in plasma tumor necrosis factor-α (TNFα) peaked at 30 minutes after LPS challenge. Up-regulation of IL-1β, IL-6 and HMGB-1 was more persistent, with detectable levels up to day three. A 15-fold increase in IL-6 and a 6.5-fold increase in IL-1β mRNA at 6 hours post intervention (*P *< 0.001 respectively) was found in the hippocampus. Reactive microgliosis was observed both at days one and three, and was associated with elevated HMGB-1 and impaired memory retention (*P *< 0.005). Preemptive administration of IL-1 receptor antagonist (IL-1Ra) significantly reduced plasma cytokines and hippocampal microgliosis and ameliorated cognitive dysfunction without affecting HMGB-1 levels. Similar results were observed in LPS-challenged mice lacking the IL-1 receptor to those seen in LPS-challenged wild type mice treated with IL-1Ra.

**Conclusions:**

These data suggest that by blocking IL-1 signaling, the inflammatory cascade to LPS is attenuated, thereby reducing microglial activation and preventing the behavioral abnormality.

## Introduction

Systemic infection produces physiological and behavioral changes both in humans and animals. The ensuing sickness behavior is characterized by a decline in cognitive function, fever, decreased food intake, somnolence, hyperalgesia, and general fatigue [1]. Most of the symptomatic effects of infection can be correlated to neuroinflammation in different brain regions, including the hippocampus [2].

Cytokines have a pivotal role in orchestrating the inflammatory response after viral or bacterial infection and are essential in restoring homeostasis. Cytokines also affect behavior, especially memory and cognition [3]. Lipopolysaccharide (LPS), comprising glycolipids from the outer membrane of Gram-negative bacteria, stimulates monocytes, macrophages, and neutrophils to produce cytokines and a plethora of other pro-inflammatory mediators. IL-1 can be considered the prototypic multifunctional and pleiotropic cytokine due to its widespread effects on immune signaling, central nervous system (CNS) functions, and its prominence in many disease states [4,5].

Learning and memory processes largely rely on the hippocampus and this brain region expresses the highest density of IL-1 receptors, making it vulnerable to the adverse consequences of neuroinflammation [6,7]. Although IL-1β is required for normal learning and memory processes, exogenous administration or excessive endogenous levels produce detrimental cognitive behavioral effects [8,9]. A synergistic interaction between IL-1β and other cytokines, such as TNFα and IL-6, enhances this cognitive dysfunction [10]. Also, other molecules, including high-mobility group box 1 (HMGB-1), have a pivotal role in the innate immune response to diseases, including sepsis [11].

Brain dysfunctions (delirium, dementia, neurodegeneration) remain a common complication in critically ill patients and are an independent risk factor for a poorer prognosis and increased mortality [12]. Various attempts have been made to target the immune system in sepsis and delirium, yet the role of cytokines and their association with cognitive dysfunctions remain poorly understood. The aim of this study is to indentify cytokines that can be targeted in order to ameliorate inflammatory-induced cognitive dysfunction following endotoxemia. Here we provide evidence that targeting the IL-1 signaling ameliorates cognitive abnormalities that does not directly depend on HMGB-1 mechanisms. The role of cytokines, in particular IL-1, and microglial activation in cognitive abnormalities is confirmed by experiments involving mice devoid of the cognate receptor (IL-1R^-/-^).

## Materials and methods

### Animals

All the experiments were conducted under the UK Home Office approved license. Wild type C57BL/6 male mice pathogen free, 12 to 14 weeks of age, weighing 25 to 30 g were housed in standard cages with no environmental enrichment in groups of five in a 12 hours light 12 hours dark cycle with controlled temperature and humidity, free access to water and standard rodent chow. IL-1R^-/- ^(kindly provided by Professor Dame Nancy Rothwell, University of Manchester [13]) were bred in-house on a C57BL/6 background and age-matched to wild type counterparts. Seven days of acclimatization were allowed before starting any experiment. All the animals were checked on a daily basis and those with evidence of poor grooming, huddling, piloerection, weight loss, back arching and abnormal activity were excluded in the experiments.

### Treatment

LPS derived from *Escherichia Coli *endotoxin (0111:B4, InvivoGen, San Diego, CA, USA, 1 mg/kg) was dissolved in normal saline and injected intraperitoneally. IL-1Ra (Amgen, Anakinra 100 mg/kg, Thousand Oaks, CA, USA) was given subcutaneously immediately before LPS administration. Dose response curve from LPS or IL-1Ra was obtained from our pilot studies to provoke or to suppress, respectively, a moderate degree of microglia activation. Control animals were injected with equivalent volumes (0.1 ml) of saline. Mice from each treatment group were randomly assigned for assessment of either cytokine response or cognitive behavior, in order to obviate possible confounding effects of behavioral testing on inflammatory markers [14].

### Plasma cytokine measurement

Blood was sampled transcardially after thoracotomy under terminal anesthesia 30 minutes, 2, 6, and 12 hours and 1, 3, and 7 days after experiments in the different cohorts and centrifuged at 3,600 rpm for 7 minutes at 4°C. Blood samples taken from animals without any interventions served as controls. Plasma samples were stored at -20°C for further analysis. Plasma cytokine and HMGB-1 were measured using commercially available ELISA kits from Biosource (Camarillo, CA, USA) and Shino-test Corporation (Kanagawa 229-0011, Japan), respectively. The sensitivities of the assays were less than 3 pg/ml for TNFα, less than 7 pg/ml for IL-1β, less than 3 pg/ml for IL-6 and 1 ng/ml for HMGB-1.

### Quantitative real time PCR

The hippocampus was rapidly extracted under a dissecting microscope, placed in RNAlater solution (Applied Biosystems, Ambion, Austin, TX, USA) and stored at 4°C. Total RNA was extracted using RNeasy Kit (Qiagen, Austin, TX, USA) and quantified. The one-step quantitative (q) PCR was performed on a Rotor-Gene 6000 (Corbett Life Science, Austin, TX, USA), using Assay-On-Demand premixed Taqman probe master mixes (Applied Biosystems, Foster City, CA, USA). Each RNA sample was run in triplicate, and relative gene expression was calculated using the comparative threshold cycle ΔΔC_t _and normalized to beta-actin. Results are expressed as fold-increases relative to controls.

### Immunohistochemistry

Mice were euthanized and perfused transcardially with ice-cold heparinized 0.1 M PBS followed by 4% paraformaldehyde in 0.1 M PBS at pH 7.4 (VWR International, Lutterworth, Leicester, UK). The brains were harvested and post-fixed in 4% paraformaldehyde in 0.1 M PBS at 4°C and cryoprotected in 0.1 M PBS solutions containing 15% sucrose for 24 hours (VWR International, Lutterworth, Leicester, UK) and then 30% sucrose for a further 48 hours. Brain tissue was freeze-mounted in optimal cutting temperature (OCT) embedding medium (VWR International, Lutterworth, Leicester, UK). The 25 μm thick coronal sections of the hippocampus were cut sequentially in groups of six and mounted on Superfrost^® ^plus slides (Menzel-Glaser, Braunschweig, Germany). The rat anti-mouse monoclonal antibody, anti-CD11b (low endotoxin, clone M1/70.15) at a concentration of 1:200 (Serotec, Oxford, UK) was used to label microglia. Visualization of immunoreactivity for CD11b was achieved using the avidin-biotin technique (Vector Labs, Cambridge, UK) and a goat anti-rat secondary antibody (Chemicon International, Temecula, CA, USA) at a concentration of 1:200. A negative control omitting the primary antibody was performed in all experiments. Immunohistochemical photomicrographs were obtained with an Olympus BX-60 microscope (Olympus Corp., Tokyo, Japan) and captured with a Zeiss KS-300 colour 3CCD camera (Carl Zeiss AG, Tokyo, Japan). The assessment of staining, by an observer that was blinded to the interventional group, was based upon a four-point categorical scale [15].

### Behavioral measurement (conditioning)

The behavioral study was conducted using a dedicated conditioning chamber (Med Associates Inc., St. Albans, VT, USA). Mice were trained and tested on separate days. LPS was injected within 30 minutes following training. The fear conditioning paradigm was used as previously described, with minor modifications [16]. Three days after training, mice were returned to the same chamber in which training occurred (context), and freezing behavior was recorded. Freezing was defined as lack of movement except that required for respiration. Approximately three hours later, freezing was recorded in a novel environment and in response to the cue (tone). The auditory cue was then presented for three minutes, and freezing scored again. Freezing scores for each subject were expressed as a percentage for each portion of the test. Memory for the context (contextual memory) for each subject was obtained by subtracting the percent freezing in the novel environment from that in the context. All assessments were performed in a blinded fashion.

### Data analysis

Statistical analyses were performed using GraphPad Prism version 5.0a (GraphPad Software, San Diego, CA, USA). The results are expressed as mean ± standard error of the mean. Data were analyzed with analysis of variance followed by Newman-Keuls *post hoc *test wherever appropriate. For categorical data, non-parametric Kruskal-Wallis followed by Dunn's test was used. A *P *< 0.05 was considered to be statistical significance.

## Results

### Endotoxin-induced cytokine production is modified by IL-1Ra and in IL-1R^-/-^

**Figure 1:**
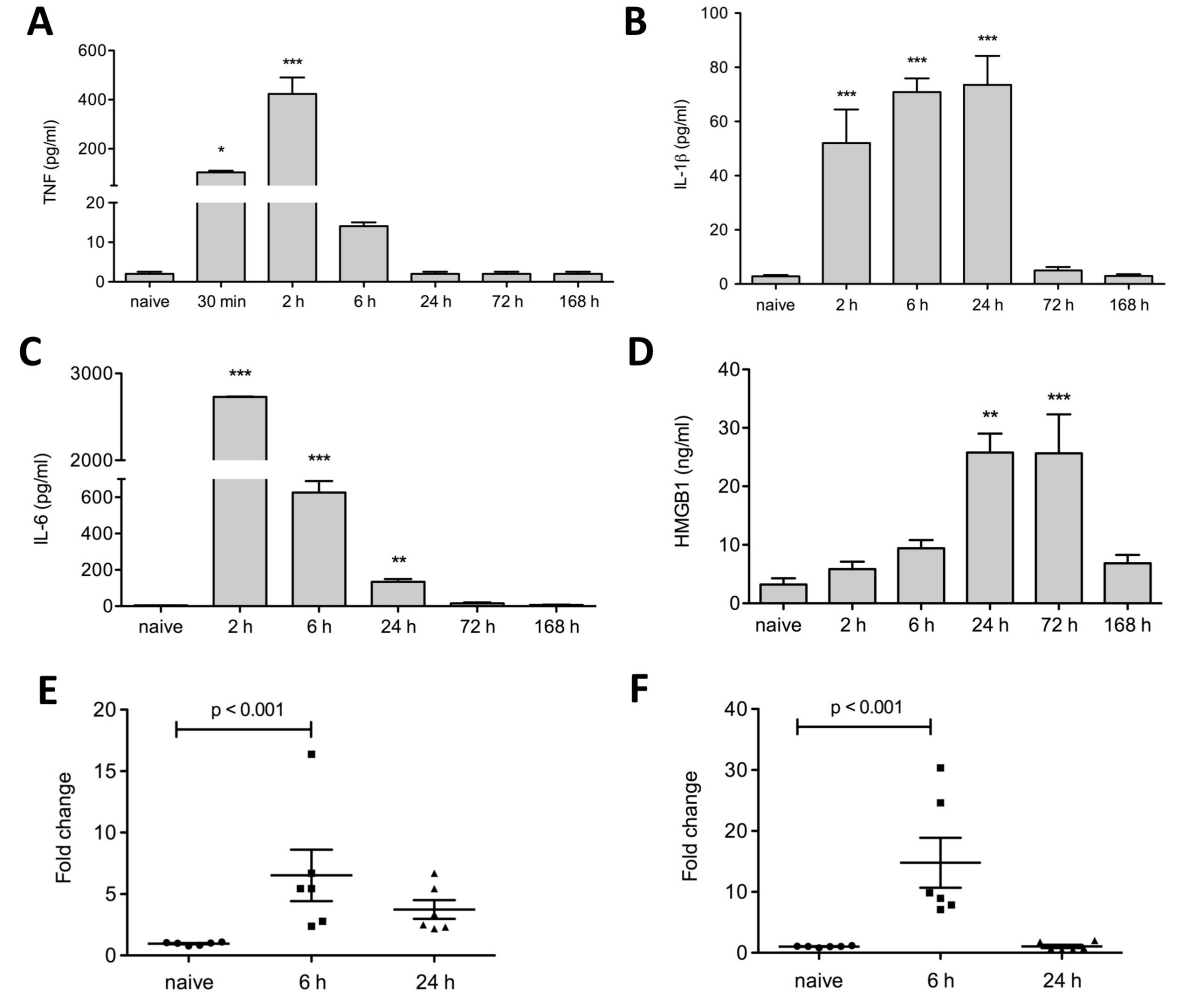
**Inflammatory response after LPS exposure**. Mice were injected with lipopolysaccharide (LPS) at time zero and plasma levels of TNFα, IL-1β, IL-6 and HMGB-1 were measured by ELISA. TNFα was increased after 30 minutes and peaked at 2 hours, returning to baseline thereafter. (a) * *P *< 0.01; *** *P *< 0.001 *vs *naïve. IL-1β was detected after two hours from LPS administration and levels continued to steadily increase until 24 hours. (b) *** *P *< 0.001 *vs *naïve. IL-6 expression was highly elevated at two hours, decreasing at six hours but still significantly detectable at 24 hours compared with naïve animals. (c) *** *P *< 0.0001; ** *P *< 0.001 *vs *naïve respectively. Levels of HMGB1 started to increase at day 1 and until day 3. (d) ** *P *< 0.001; *** *P *< 0.0001 *vs *naïve. Increased mRNA expression of **(e) **IL-1β and **(f) **IL-6 was found at six hours after peripheral LPS injection in the hippocampus of mice using quantitative PCR (*P *< 0.001 *vs *naïve); mRNA expression returned to normal by day 1. Data are expressed as mean ± standard error of the mean (n = 6) and compared by one-way analysis of variance and Student-Newman-Keuls method.

**Figure 2:**
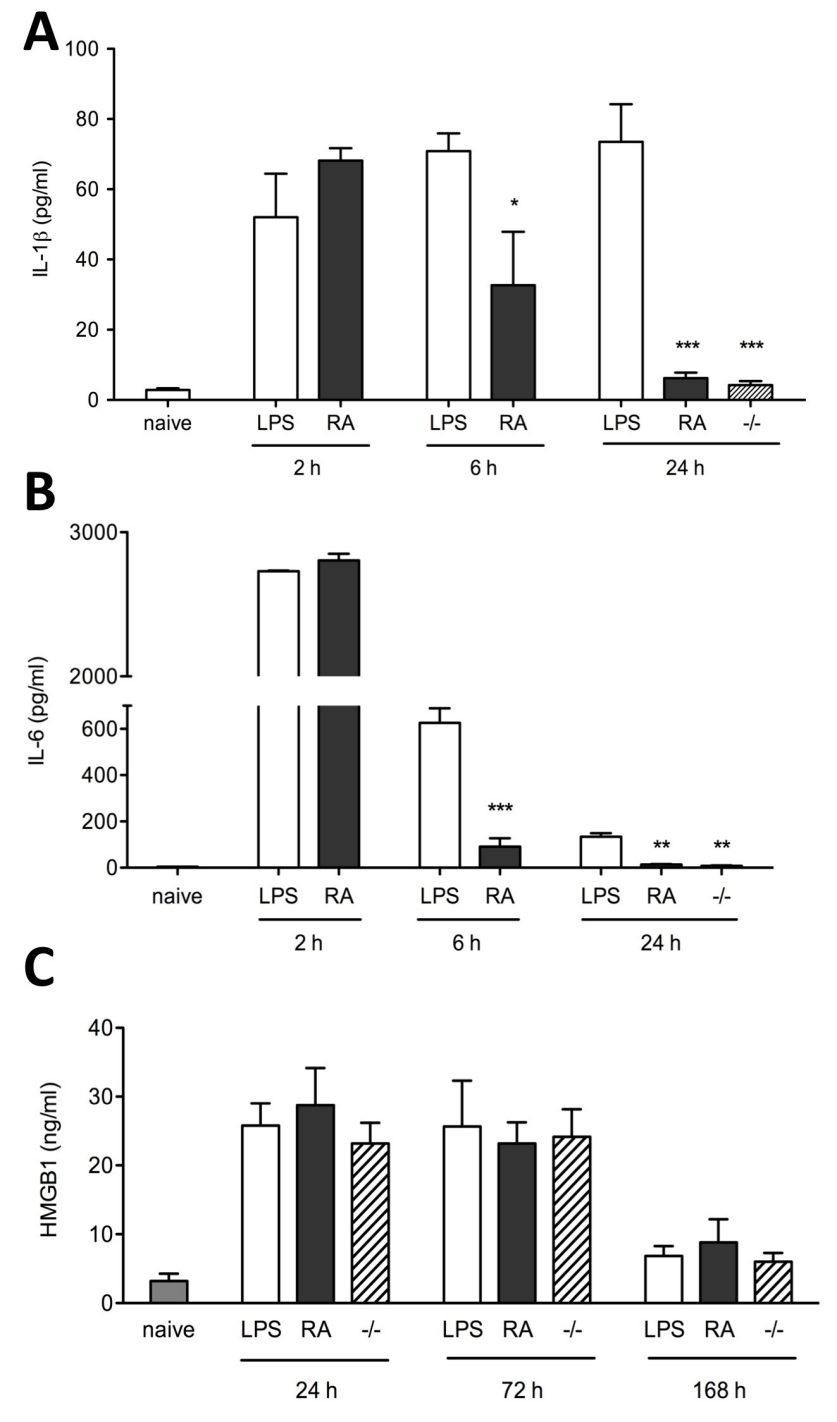
**Blocking IL-1 reduces systemic cytokine release**. Animals received lipopolysaccharide (LPS) or treatment with IL-1Ra immediately before LPS exposure (RA). Plasma levels of IL-1β and IL-6 were measured by ELISA at 2, 6, and 24 hours. Pre-emptive administration of IL-1Ra significantly reduced the amount of plasma IL-1β at six hours (a * *P *< 0.01 *vs *LPS) and 24 hours (*** *P *< 0.001 *vs *LPS). IL-6 followed a similar trend, with a strong decrease in plasma concentrates at six hours (b *** *P *< 0.001 *vs *LPS) and at 24 hours (** *P *< 0.001 *vs *LPS). To corroborate the findings, levels of IL-1β and IL-6 were measured in IL-1R^-/- ^(-/-) (**a to b**, *** *P *< 0.0001 and ** *P *< 0.001 *vs *LPS respectively). **(c) **IL-1Ra or IL-1R^-/- ^had no effects on HMGB-1 release in plasma. Data are expressed as mean ± standard error of the mean (n = 6) and compared by one-way and two-way (IL1R^-/-^) analysis of variance and Student-Newman-Keuls method.

To investigate the effects of inflammation on cognitive function we measured systemic and central cytokines after LPS administration. TNFα release occurred very rapidly and transiently; after 30 minutes it was significantly increased (104.18 ± 7.36 pg/ml), peaking at two hours and returning to normal at six hours post-injection (Figure 1a; *P *< 0.01, *P *< 0.001 vs control). LPS evoked a robust systemic response that induced a stereotypical cytokine release. Both IL-1β and IL-6 were significantly up-regulated from two hours. IL-1β increased four-fold and plasma levels continued to steadily increase until 24 hours (Figure 1b; 73.49 ± 5.42 pg/ml, *P *< 0.001 vs control). IL-6 expression was markedly elevated at two hours, decreasing at six hours but still significantly detectable at 24 hours compared with naïve animals (Figure 1c; 134.37 ± 8.43 pg/ml, *P *< 0.01 vs control). During this time, animals showed classic symptoms of sickness behavior (reduced motility, poor grooming, huddling, piloerection, back arching). Levels of HMGB-1 at 2, 6, and 12 hours post LPS were no different from baseline levels; a 1.5-fold increase was observed from 24 hours after LPS and remained elevated up to day 3 (Figure 1d; 25.77 ± 4.2 pg/ml, *P *< 0.01, *P *< 0.001 vs control). The systemic inflammatory response resolved after day three and all cytokine levels returned to baseline by day seven. To assess the central inflammatory response to LPS we measured levels of IL-1β and IL-6 mRNA expression in the hippocampus. We noted a 6.5-fold increase in IL-1β mRNA expression and a 15-fold increase in IL-6 in the hippocampus at six hours after LPS injection (Figures 1e and 1f; *P *< 0.001 vs control). In both cases the increased transcription returned to normal values by 24 hours. The increase in IL-1β both in plasma and in the hippocampus led us to investigate whether blocking the IL-1 receptor could ameliorate the signs of LPS-associated cognitive dysfunction. A single preemptive dose of IL-1 Ra was able to significantly reduce plasma levels of IL-1β at 6 and 24 hours (Figure 2a, 32.7 ± 5.45 pg/ml, 6.2 ± 1.03 pg/ml, *P *< 0.01 and *P *< 0.001 vs LPS, respectively). Similarly, levels of IL-6 were also reduced at the same time-points (Figure 2b; 91.02 ± 15.17 pg/ml, 14.05 ± 2.34 pg/ml, *P *< 0.001, *P *< 0.001 vs LPS, respectively). Interestingly, IL-1Ra treatment had no effects on HMGB-1 levels, which maintained a similar pattern to that seen after LPS injection in the absence of IL-1Ra (Figure 2c).

Corroboration of these data was achieved by injecting IL-1R^-/- ^animals with the same dose of LPS and measuring cytokine expression in plasma. At 24 hours, after LPS in the IL-1R^-/-^, a time at which there was markedly increased cytokines and clear evidence of sickness behavior in untreated wild type mice, levels of IL-1β and IL-6 were comparable with the wild type mice that received IL-1Ra treatment (Figures 2a and 2b; *P *< 0.0001, *P *< 0.001 vs LPS). Contrary to the cytokine changes, the LPS-induced elevation of HMGB-1 was not abrogated in the IL-1R^-/- ^or IL-1Ra-treated animals (Figure 2c).

### LPS-induced microglial activation is modified by IL-1Ra and absent in IL-1R^-/-^

**Figure 3:**
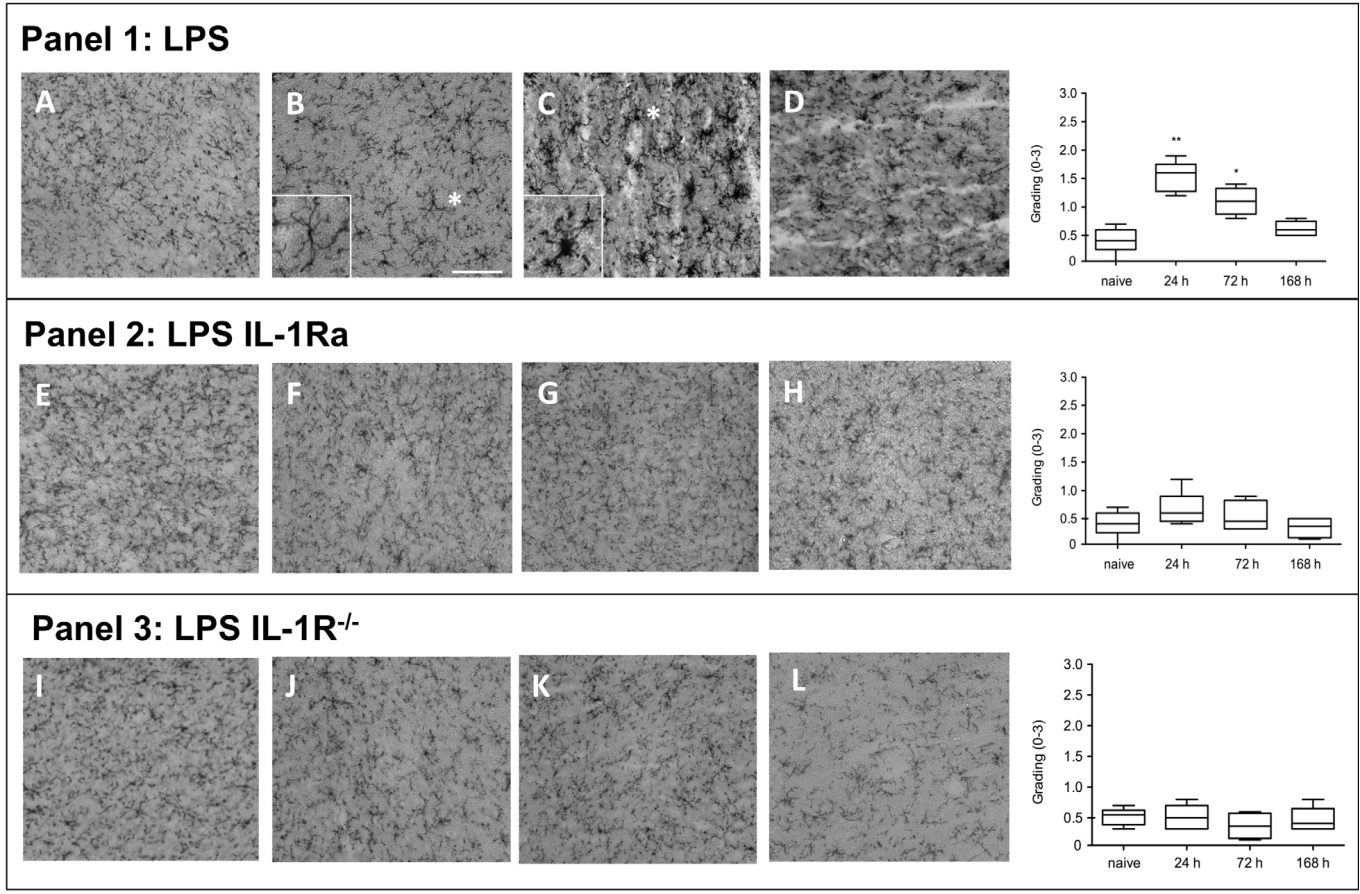
**Blocking IL-1 reduces microglia activation**. Hippocampi were harvested at days 1, 3, and 7 after lipopolysaccharide (LPS) administration and stained with anti-CD11b. Pictures show CA1 (scale bar 50 μm, 20×) and photomicrographs were blindly scored and microglia activation was graded on a scale 0 (lowest) to 3 (highest). **(a, e and i) **normal controls; no microgliosis was observed in wild type nor IL-1R^-/-^. **Panel 1: LPS**. Reactive microglia were found at days 1 and 3 **(c and d) **after LPS injection compared with **(b) **naive. Resting microglia (box a, 40×) shifted to a 'reactive state' (box b, 40×). **Panel 2: IL-1Ra**. Reduction in the number of reactive microglia was observed **(g and h) **after administering IL-1Ra both at days 1 and 3, **(f) **with no changes from controls. **Panel 3: IL-1R**^-/-^. **(j to l) **Administration of LPS to IL-1R^-/- ^did not induce microglia activation at any time point assessed. Immunohistochemical grading (0 to 3) illustrates panels 1, 2, and 3. One day after LPS administration we found clear microgliosis, which was attenuated by IL-1Ra treatment (day 1 ** *P *< 0.001 *vs *naïve, day 3 * *P *< 0.05 *vs *naïve). Significant reduction in microgliosis was found both after IL-1 Ra administration and in IL-1R^-/- ^(n = 4). Non parametric data are presented with Kruskal-Wallis followed by Dunn's test.

The hippocampal transcriptome findings of the pro-inflammatory cytokines prompted interest for other possible markers of neuroinflammation. Normal controls, both from WT and IL-1R^-/-^, showed no signs of microgliosis (Figures 3a, e and 3i). Minimal immunoreactivity was reported in naïve animals in which microglia maintained small cell bodies with thin and long ramified pseudopodia (Figure 3b). Resting microglia shifted to a 'reactive profile' after LPS exposure, acquiring an amoeboid morphology with hypertrophy of the cell body and retraction of the pseudopodia. Reactive microglia displayed morphological changes including increased cell body dimensions, shortened and clumpy processes with higher levels of CD11b immunoreactivity compared with naïve animals. Microglial activation was reported at days one and three post exposure (Figures 3c and d;*P *< 0.01, *P *< 0.05 vs control, respectively), returning to the baseline resting state by day seven. Pre-treatment with IL-1Ra effectively reduced the number of reactive microglia at days one and three (Figures 3f and 3g). In order to corroborate these findings, we repeated the experiment using IL-1R^-/- ^animals and exposing them to LPS. No microglial activation was noted in LPS treated IL-1R^-/- ^mice (Figures 3k and 3l).

### Hippocampal-dependent cognitive dysfunction following LPS is ameliorated by IL-1 blockade

**Figure 4:**
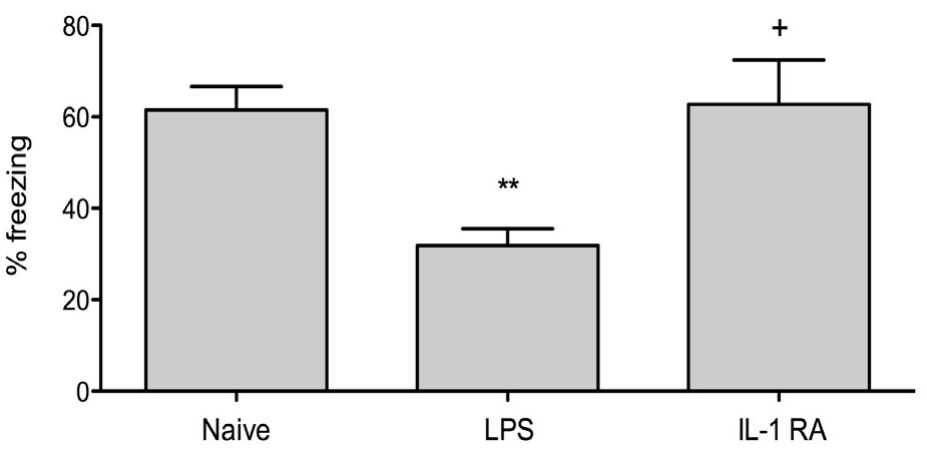
**Contextual fear response is ameliorated by pre-emptive IL-1 Ra**. Within 30 minutes following training, mice were injected with lipopolysaccharide (LPS). Three days later, rodents were exposed to the same context in which fear conditioning was previously carried out. Contextual fear response reveals a clear hippocampal-dependent memory impairment (** *P *< 0.005 *vs *naive). Pre-treatment with IL-1Ra abolished the main symptoms of sickness behavior and significantly ameliorated the memory retention at day 3 (+ *P *< 0.05 *vs *LPS). Data are expressed as mean ± standard error of the mean (n = 9 for acute behavior) and compared by one-way analysis of variance and Student-Newman-Keuls method.

**Figure 5:**
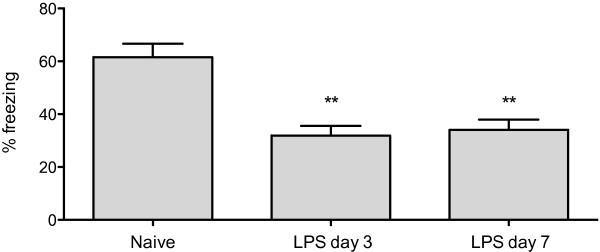
**Contextual fear response following LPS administration**. Thirty minutes after undergoing contextual fear conditioning mice received LPS injection. Three and seven days later rodents were exposed to the same context in which fear conditioning was previously carried out. Contextual fear response reveals a clear hippocampal-dependent memory impairment both at day 3 and 7 (** *P *< 0.005 *vs *naive). Data are expressed as mean ± standard error of the mean (n = 6 for acute behavior) and compared by one-way analysis of variance and Student-Newman-Keuls method.

To relate the inflammatory response to memory functioning, we used trace fear conditioning in which mice are trained to associate a tone with a noxious stimulation (foot shock). The brief gap between the tone termination and the shock onset allows assessment of hippocampal integrity [16]. The high level of freezing seen in the naïve animals is indicative of good learning and memory retention. Contextual fear response shows a reduced immobility (freezing) at day three, revealing and hippocampal-dependent memory impairment (Figure 4; *P *< 0.005 vs naïve trained). Pre-treatment with IL-1Ra significantly ameliorated this cognitive dysfunction, abolishing also the symptoms of sickness behaviour otherwise evident in LPS-treated animals (Figure 4; *P *< 0.05 vs LPS). During the initial 24 hours following LPS administration animals show classic sign of sickness behavior, in particular reduced motility, poor grooming, and back arching. Remarkably, animals treated with pre-emptive IL-1Ra had no signs of sickness behavior, which functionally reflected in better memory retention and no microgliosis. LPS administration caused a permanent retrograde amnesia at both days 3 and 7 (Figure 5).

## Discussion

These data show that a sustained inflammatory challenge leads to neuroinflammation, microglial activation and hippocampal-mediated cognitive dysfunction. By blocking the IL-1 receptor, the feed-forward process that amplifies the inflammatory cascade is attenuated thereby reducing microglial activation and reversing the behavioral abnormality after endotoxemia.

### Peripheral and central cytokines contribute to the inflammatory milieu in sickness behavior

Cytokines play an important role in mediating the inflammatory response after infection or aseptic traumatic injury. The innate immunity is rapidly triggered after LPS, primarily via activation of toll-like receptor 4 (TLR-4) [17]. Activation of TLR-4 induces a multitude of pro-inflammatory cytokines via activation of transcription factors, nuclear factor (NF)κB [18]. This prompt response provides a favorable environment for the synthesis and upregulation of both IL-1β and IL-6, which together contribute to the perpetuation of the inflammatory challenge. Also the rapid increase in TNFα following LPS, which is reported as already present after 30 minutes, promotes synthesis of other cytokines and the initiation of the acute-phase response, chemokine release and oxidative stress. Systemic cytokines, including IL-1β, can bind receptors and translocate through the intact blood-brain barrier (BBB) [19]. Neural afferents are known to be a fast and reliable pathway in the immune-to-brain signaling. Vagal-mediated signaling can rapidly induce brain cytokines and manifest the classic symptoms of the acute-phase response, including neuroinflammation [20]. As we have reported a significant increase in both IL-1β and IL-6 mRNA transcription at six hours in the hippocampus, the neuronal route may be the likely pathway to trigger the early activation of these genes and the initial changes in the CNS. Vagotomy was previously shown to partially attenuate sickness behavior both after LPS and IL-1β administration [21], but not in the context of hippocampal-dependent cognitive dysfunction.

### Reactive microglia in the hippocampus interfere with memory processing

Within the brain, cytokines interact with microglia cells. Pro-inflammatory cytokines can directly interact with many of the pattern recognition receptors expressed on the surface of these cells [22]. Upon activation, microglia exhibit discernible morphologic changes and secrete cytokines, reactive oxygen species, excitotoxins (such as calcium and glutamate) and neurotoxins such as amyloid-β [23]. Activated microglia also inhibit neurogenesis in the hippocampus following endotoxemia, thereby exacerbating the extent of injury on memory processing [24].

To assess memory retention we used trace fear conditioning in which mice are trained to associate a foot shock with a given environment or tone [25]. The extent to which an animal freezes to a context is largely dependent on the hippocampus [26]. Hippocampal-dependent memory impairment was evident after three days post-LPS. Residual inflammation, primarily via reactive microglia, is possibly associated with this second-phase behavioral abnormality. At these time points, levels of HMGB-1 were also elevated and prompted us to further investigate the role of these factors in the development of cognitive dysfunction. As the cognitive impairment was also present at day seven post LPS exposure (Figure 5) when inflammatory markers returned to baseline, this suggests that the initial acute-phase response may have interfered with processes of memory consolidation in the hippocampus.

### Targeting IL-1 ameliorates the cognitive abnormality by reducing microglia but does not affect HMGB1

IL-1β has a pivotal role in sustaining the neuroinflammatory response and closely interacts with memory processing and long-term potentiation [27,28]. Self-regulation and inhibition of IL-1β is normally achieved with the neutralizing action of endogenous IL-1Ra, which directly competes for binding to the receptor [29,30]. Transcription of endogenous IL-1Ra would normally occur temporally delayed from the synthesis of IL-1, thus following pharmacological intervention we aimed to block the receptor *a priori *impeding binding and limiting the damage mediated by the effector molecule. When the IL-1 receptor is disabled, either blocked pharmacologically (IL-1Ra) or by genetic intervention (IL-1R^-/-^), the inflammatory response is not sustained as reflected by lower cytokine release and microglia activation, thus ameliorating the cognitive dysfunction as reported here. Treatment with IL-1Ra provides a significant improvement in cognitive dysfunction, confirming the crucial role of IL-1β in memory processes and behavior. However, as IL-1Ra exerts protective effects also by reducing apoptosis and ischemia [31], the behavioral improvement could also reflect a wider action of this treatment not only on the immune system. Although there was a temporal correlation between microglia activation and late-release of HMGB-1, neither IL-1Ra nor IL-1R^-/- ^changed levels of this cytokine. This evidence supports the notion that blocking IL-1 is sufficient to reduce the microglia activation and ameliorate the memory abnormality. Other receptors may be involved in sustaining this inflammatory challenge; for example, HMGB-1 has been shown to activate TLRs and receptor for advanced glycation end-products and it has been reported as a key late pro-inflammatory mediator in sepsis, with considerable pathological potential [11,32].

Some limitations of our study must be pointed out. As IL-1Ra is able to translocate directly into the brain [33], we are unable to discriminate whether peripheral cytokines and/or *de-novo *production in the CNS account for this cognitive dysfunction. Also, recently it has been shown that peripheral monocytes can enter the brain causing sickness behavior. This process strongly relies on TNFα signaling, especially in activating microglia and recruiting active monocytes into the CNS [34]. By targeting microglia we have selected a robust marker to correlate local inflammation with the functional behavioral abnormality. However, in this study we cannot determine the nature of the microgliosis, whether they are infiltrated macrophages that crossed the BBB or actual microglia. Although our primary aim was to characterize the importance of inflammatory mediators in cognitive dysfunction and by using LPS this can be more easily defined, a septic model using cecal ligation and perforation would have been more clinically applicable in reproducing the complexity of the polymicrobial septic pathology.

## Conclusions

The beneficial effects on cognition reported in this study by targeting IL-1, preemptively, are encouraging. However, it is not possible to extrapolate these benefits to the setting of cognitive dysfunction that accompanies severe sepsis with multiple organ failure. In that clinical scenario there are complex inflammatory responses, various humoral factors, oxidative stress, acid-base and hemodynamic dysfunctions that are difficult to reverse [35]. Using LPS we have selected a well-defined stimulus for the innate immunity, which has enabled to better identify key molecules and pathways in LPS-induced cognitive dysfunction. These data now prompt us to further investigate these therapies using established models of sepsis and multiple organ failure. Clinical trials targeting IL-1 have been unconvincing in improving mortality rate, especially in sepsis [36]. In this attempt to untangle the complexity of this condition, anti-IL-1 therapy appears to be able to ameliorate the associated cognitive dysfunction, independently of other mechanisms. Inflammation clearly plays a pivotal role in mediating physiological as well as behavioral changes after LPS-exposure. Further studies are needed to ascertain whether selective targeting of other cytokine receptors can effectively prevent or ameliorate both the degree and length of cognitive decline.

## Key messages

• Neuroinflammation plays a pivotal role in mediating physiological and behavioral changes after LPS.

• Up-regulation of microglia and HMGB-1 correlates in a temporal fashion with the cognitive dysfunction.

• Blocking IL-1 does not affect HMGB-1 release; however, it reduces microglia activation reversing the behavioral abnormality.

• In the absence of IL-1, HMGB-1 is insufficient to sustain hippocampal neuroinflammation and the attendant cognitive dysfunction. Further studies are required to investigate the potential benefit of anti-cytokine therapy in the ICU.

## Abbreviations

BBB: blood-brain barrier; CNS: central nervous system; ELISA: enzyme-linked immunosorbent assay; HMGB-1: high-mobility group box 1; IL: interleukin; LPS: lipopolysaccharide; NF: nuclear factor; PBS: phosphate-buffered saline; qPCR: quantitative polymerase chain reaction; TLR: toll-like receptor; TNFα: tumor necrosis factor-α.

## Competing interests

In aseptic trauma-induced cognitive dysfunction, we have identified a therapeutic intervention for which a patent has been applied. This is unrelated to sepsis-induced cognitive dysfunction.

## Authors' contributions

The hypothesis was developed by NT in conjunction with MM, CM, DM, MV and MF. All authors contributed to the study design and interpretation. NT, AF, and MC performed the experiments. NT drafted the manuscript with MM, CM, and DM. NT and AF contributed equally to the paper. All authors reviewed the manuscript and contributed to editing it for publication.
